# Draft Genome Sequence of the Community-Associated Staphylococcus aureus Sequence Type 88 Strain LVP-7, Isolated from an Ocular Infection

**DOI:** 10.1128/MRA.00077-21

**Published:** 2021-02-18

**Authors:** Savitha Nadig, Sneha Murthy, Muralidharan Vandanashree, Hosahalli S. Subramanya, Balasubramanian Gopal, Shruthi Vembar

**Affiliations:** a Division of Biological Sciences, Indian Institute of Science, Bangalore, India; b The University of Trans-Disciplinary Health Sciences and Technology, Bangalore, India; c Institute of Bioinformatics and Applied Biotechnology, Bangalore, India; University of Rochester School of Medicine and Dentistry

## Abstract

We report a *de novo*-assembled draft genome sequence of the Indian Staphylococcus aureus sequence type 88 (ST88) strain LVP-7, isolated from an ocular infection. The genome harbors a Panton-Valentine leukocidin phage, a type V staphylococcal cassette chromosome *mec* element, the delta-hemolysin-converting Newman phage ΦNM3, and the pathogenicity island SaPI3, encoding the superantigen enterotoxin B.

## ANNOUNCEMENT

Staphylococcus aureus is among the most common pathogens causing ocular infection. Previous reports suggest that both hospital-associated (HA) and community-associated (CA) methicillin-resistant Staphylococcus aureus (MRSA) can cause eye infections (1–3). In India, CA MRSA and methicillin-sensitive *Staphylococcus aureus* (MSSA) strains belong to distinct clonal lineages carrying either type IV or V staphylococcal cassette chromosome *mec* (SCC*mec*) elements (4, 5). The sequence type 88 (ST88) lineage is more prevalent in India (4, 6), Africa (3, 7), and China (8), unlike other Indian CA-MRSAs such as ST772 that are globally disseminated (9–11).

Here, we report a draft genome sequence of an ST88 CA MRSA strain. Strain LVP-7 was isolated from an orbital abscess in a patient at LV Prasad Eye Institute, Bhubaneswar, India. This isolate is not part of a larger epidemiological study and is exempt from ethics committee approval. For genomic DNA (gDNA) extraction, LVP-7 glycerol stock stored at −80°C was streaked onto chromogenic agar medium (chromAgar, bioMérieux, Marcy-L’Etoile, France). A single colony was picked and grown overnight in brain heart infusion (BHI) broth under aerobic conditions. gDNA was prepared using the phenol-chloroform method (4). Sequencing libraries were prepared using the NEBNext DNA Ultra II library prep kit (New England Biolabs) and sequenced using v3 chemistry in an Illumina HiSeq 2500 instrument (2 × 100-bp paired-end format). A total of 9,561,330 read pairs were demultiplexed to fastq format using bcl2fastq v2.20.0.422. The quality of the fastq files was ascertained using FastQC v0.11.7 (12). Adapter content and low-quality reads were removed using Trim Galore (13). *De novo* assembly was performed using SPAdes v3.14.1 (14) and assembly quality assessed using QUAST v4.5 (15). Gap filling, ordering of contigs, and optimal scaffolding were done using RagTag (16), with the S. aureus M013 genome as reference (17). The resulting assembly was annotated using PROKKA v1.14.6 (18), and downstream analyses were performed using SCC*mec*Finder, SPAtyper v0.1.0, and TA finder (19–21). The NAuRA-curated enterotoxin database (22) was used to predict toxin gene clusters. ResFinder and PathogenFinder were used to identify the antibiotic resistance and virulence gene clusters, respectively (23, 24). Prophage Hunter and PhiSpy helped to identify prophage gene signatures (25, 26). Default parameters were used for all software unless otherwise specified. Details of the assembled genome sequence and annotation are compiled in Table 1.

**TABLE 1:** Genome assembly statistics and annotation features of the Staphylococcus aureus ST88 strain LVP-7

Feature	Value
Draft *de novo* assembly statistics	
No. of contigs	69
No. of contigs >500 bp	16
Largest contig size (bp)	2,777,888
Genome size (bp)	2,858,759
G+C content (%)	32.73
*N*_50_ (bp)	2,777,888
No. of *N*s per 100 kbp	217.54
Genome annotation features	
No. of ORFs^*a*^	2,722
No. of mRNAs and rRNAs	2,668
No. of tRNAs	53
No. of tmRNAs^*b*^	1
Positive strand (bp)	1,323
Negative strand (bp)	1,399

a ORFs, open reading frames.

b tmRNAs, transfer-messenger RNAs.

**FIG 1:**
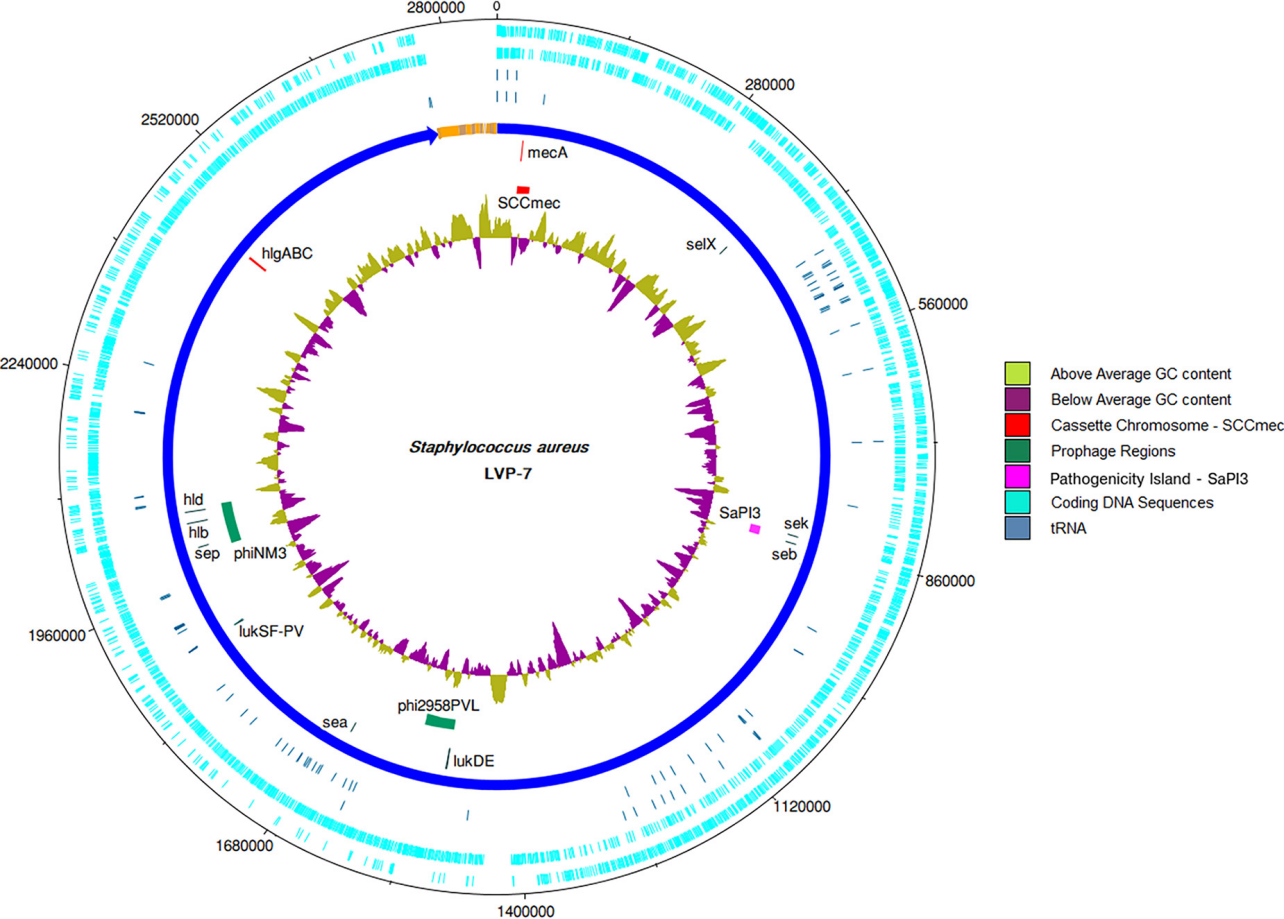
Major genetic elements in Staphylococcus aureus ST88 strain LVP-7. The innermost circular track (track 1) represents the G+C content. Track 2 displays select mobile genetic elements, including the staphylococcal cassette chromosome (SCC*mec*) element, the pathogenicity island SaPI3, and the Panton-Valentine leukocidin (PVL) and phiNM3 prophages. Track 3 displays select virulence genes. While track 4 represents the major (blue) and minor (orange) contigs, tracks 5 and 6 show the location of the tRNAs. The outer tracks (7 and 8) represent coding sequences. This representation was made using DNAPlotter (29).

The draft genome sequence reveals that LVP-7 belongs to the *spa* (Staphylococcus aureus protein A) type t2526 and carries an SCC*mec* type V (5C2) cassette. This ST88 strain encodes Panton-Valentine leukocidin (PVL) phage Φ2958PVL, gamma-hemolysin components (hlgABC), and several super antigens such as *sea*, *sep*, *sek*, and *selX* (Fig. 1). A δ-hemolysin (*hlb*)-converting S. aureus Newman phage (ΦNM3) was also identified, as depicted in Fig. 1. The staphylococcal pathogenicity island (SaPI3; Fig. 1) in LVP-7 harbors the *seb* enterotoxin, which may contribute to systemic S. aureus infection (27). The accessory gene regulator (agr) quorum-sensing system in LVP-7 is part of *agr* allele group III and was confirmed using multiplex PCR (28). The role of these virulence genes in LVP-7 pathogenesis needs further assessment.

### Data availability.

This whole-genome shotgun project has been deposited at DDBJ/ENA/GenBank under the accession number JADRJK000000000. The associated BioProject and BioSample accession numbers are PRJNA679674 and SAMN16843707, respectively. The raw reads from Illumina sequencing have been submitted to the Sequence Read Archive (SRA) and are available under the accession number PRJNA679674.

## References

[1] Ramesh S, Ramakrishnan R, Bharathi MJ, Amuthan M, Viswanathan S 2010 Prevalence of bacterial pathogens causing ocular infections in South India. Indian J Pathol Microbiol 53:281–286. doi:10.4103/0377-4929.64336. 20551533

[2] Hesje CK, Sanfilippo CM, Haas W, Morris TW 2011 Molecular epidemiology of methicillin-resistant and methicillin-susceptible *Staphylococcus aureus* isolated from the eye. Curr Eye Res 36:94–102. doi:10.3109/02713683.2010.534229. 21158584 PMC3021952

[3] Ghebremedhin B, Olugbosi MO, Raji AM, Layer F, Bakare RA, Konig B, Konig W 2009 Emergence of a community-associated methicillin-resistant *Staphylococcus aureus* strain with a unique resistance profile in Southwest Nigeria. J Clin Microbiol 47:2975–2980. doi:10.1128/JCM.00648-09. 19571020 PMC2738091

[4] Nadig S, Velusamy N, Lalitha P, Kar S, Sharma S, Arakere G 2012 *Staphylococcus aureus* eye infections in two Indian hospitals: emergence of ST772 as a major clone. Clin Ophthalmol 6:165–173. doi:10.2147/OPTH.S23878. 22291460 PMC3267539

[5] Shambat S, Nadig S, Prabhakara S, Bes M, Etienne J, Arakere G 2012 Clonal complexes and virulence factors of *Staphylococcus aureus* from several cities in India. BMC Microbiol 12:64. doi:10.1186/1471-2180-12-64. 22548694 PMC3438138

[6] Chakrakodi B, Prabhakara S, Nagaraj S, Etienne J, Arakere G 2014 High prevalence of ciprofloxacin resistance in community associated *Staphylococcus aureus* in a tertiary care Indian hospital. Adv Microbiol 4:133–141. doi:10.4236/aim.2014.42018.

[7] Kpeli G, Buultjens AH, Giulieri S, Owusu-Mireku E, Aboagye SY, Baines SL, Seemann T, Bulach D, Goncalves da Silva A, Monk IR, Howden BP, Pluschke G, Yeboah-Manu D, Stinear T 2017 Genomic analysis of ST88 community-acquired methicillin resistant *Staphylococcus aureus* in Ghana. PeerJ 5:e3047. doi:10.7717/peerj.3047. 28265515 PMC5333547

[8] Liu Y, Wang H, Du N, Shen E, Chen H, Niu J, Ye H, Chen M 2009 Molecular evidence for spread of two major methicillin-resistant *Staphylococcus aureus* clones with a unique geographic distribution in Chinese hospitals. Antimicrob Agents Chemother 53:512–518. doi:10.1128/AAC.00804-08. 19029328 PMC2630620

[9] Prabhakara S, Khedkar S, Loganathan RM, Chandana S, Gowda M, Arakere G, Seshasayee ASN 2012 Draft genome sequence of *Staphylococcus aureus* 118 (ST772), a major disease clone from India. J Bacteriol 194:3727–3728. doi:10.1128/JB.00480-12. 22740659 PMC3393495

[10] Balakuntla J, Prabhakara S, Arakere G 2014 Novel rearrangements in the staphylococcal cassette chromosome mec type V elements of Indian ST772 and ST672 methicillin resistant *Staphylococcus aureus* strains. PLoS One 9:e94293. doi:10.1371/journal.pone.0094293. 24722327 PMC3983117

[11] Steinig EJ, Andersson P, Harris SR, Sarovich DS, Manoharan A, Coupland P, Holden MTG, Parkhill J, Bentley SD, Robinson DA, Tong SYC 2015 Single-molecule sequencing reveals the molecular basis of multidrug-resistance in ST772 methicillin-resistant *Staphylococcus aureus*. BMC Genomics 16:388. doi:10.1186/s12864-015-1599-9. 25981586 PMC4432960

[12] Andrews S, Krueger F, Segonds-Pichon A, Biggins L, Krueger C, Wingett S 2010 FastQC: a quality control tool for high throughput sequence data. https://www.bioinformatics.babraham.ac.uk/projects/fastqc.

[13] Bolger AM, Lohse M, Usadel B 2014 Trimmomatic: a flexible trimmer for Illumina sequence data. Bioinformatics 30:2114–2120. doi:10.1093/bioinformatics/btu170. 24695404 PMC4103590

[14] Bankevich A, Nurk S, Antipov D, Gurevich AA, Dvorkin M, Kulikov AS, Lesin VM, Nikolenko SI, Pham S, Prjibelski AD, Pyshkin AV, Sirotkin AV, Vyahhi N, Tesler G, Alekseyev MA, Pevzner PA 2012 SPAdes: a new genome assembly algorithm and its applications to single-cell sequencing. J Comput Biol 19:455–477. doi:10.1089/cmb.2012.0021. 22506599 PMC3342519

[15] Gurevich A, Saveliev V, Vyahhi N, Tesler G 2013 QUAST: quality assessment tool for genome assemblies. Bioinformatics 29:1072–1075. doi:10.1093/bioinformatics/btt086. 23422339 PMC3624806

[16] Alonge M, Soyk S, Ramakrishnan S, Wang X, Goodwin S, Sedlazeck FJ, Lippman ZB, Schatz MC 2019 RaGOO: fast and accurate reference-guided scaffolding of draft genomes. Genome Biol 20:224. doi:10.1186/s13059-019-1829-6. 31661016 PMC6816165

[17] Huang T-W, Chen F-J, Miu W-C, Liao T-L, Lin A-C, Huang I-W, Wu K-M, Tsai S-F, Chen Y-T, Lauderdale T-LY 2012 Complete genome sequence of *Staphylococcus aureus* M013, a pvl-positive, ST59-SCCmec type V strain isolated in Taiwan. J Bacteriol 194:1256–1257. doi:10.1128/JB.06666-11. 22328755 PMC3294775

[18] Seemann T 2014 Prokka: rapid prokaryotic genome annotation. Bioinformatics 30:2068–2069. doi:10.1093/bioinformatics/btu153. 24642063

[19] Kaya H, Hasman H, Larsen J, Stegger M, Johannesen TB, Allesoe RL, Lemvigh CK, Aarestrup FM, Lund O, Larsen AR 2018 SCCmecFinder, a Web-based tool for typing of staphylococcal cassette chromosome mec in *Staphylococcus aureus* using whole-genome sequence data. mSphere 3:e00612-17. doi:10.1128/mSphere.00612-17. 29468193 PMC5812897

[20] Harmsen D, Claus H, Witte W, Rothganger J, Claus H, Turnwald D, Vogel U 2003 Typing of methicillin-resistant *Staphylococcus aureus* in a university hospital setting by using novel software for spa repeat determination and database management. J Clin Microbiol 41:5442–5448. doi:10.1128/jcm.41.12.5442-5448.2003. 14662923 PMC309029

[21] Xie Y, Wei Y, Shen Y, Li X, Zhou H, Tai C, Deng Z, Ou H-Y 2018 TADB 2.0: an updated database of bacterial type II toxin-antitoxin loci. Nucleic Acids Res 46:D749–D753. doi:10.1093/nar/gkx1033. 29106666 PMC5753263

[22] Merda D, Felten A, Vingadassalon N, Denayer S, Titouche Y, Decastelli L, Hickey B, Kourtis C, Daskalov H, Mistou M-Y, Hennekinne J-A 2020 NAuRA: genomic tool to identify staphylococcal enterotoxins in *Staphylococcus aureus* strains responsible for foodborne outbreaks. Front Microbiol 11:1483. doi:10.3389/fmicb.2020.01483. 32714310 PMC7344154

[23] Bortolaia V, Kaas RS, Ruppe E, Roberts MC, Schwarz S, Cattoir V, Philippon A, Allesoe RL, Rebelo AR, Florensa AF, Fagelhauer L, Chakraborty T, Neumann B, Werner G, Bender JK, Stingl K, Nguyen M, Coppens J, Xavier BB, Malhotra-Kumar S, Westh H, Pinholt M, Anjum MF, Duggett NA, Kempf I, Nykasenoja S, Olkkola S, Wieczorek K, Amaro A, Clemente L, Mossong J, Losch S, Ragimbeau C, Lund O, Aarestrup FM 2020 ResFinder 4.0 for predictions of phenotypes from genotypes. J Antimicrob Chemother 75:3491–3500. doi:10.1093/jac/dkaa345. 32780112 PMC7662176

[24] Cosentino S, Voldby Larsen M, Moller Aarestrup F, Lund O 2013 PathogenFinder—distinguishing friend from foe using bacterial whole genome sequence data. PLoS One 8:e77302. doi:10.1371/journal.pone.0077302. 24204795 PMC3810466

[25] Song W, Sun H-X, Zhang C, Cheng L, Peng Y, Deng Z, Wang D, Wang Y, Hu M, Liu W, Yang H, Shen Y, Li J, You L, Xiao M 2019 Prophage Hunter: an integrative hunting tool for active prophages. Nucleic Acids Res 47:W74–W80. doi:10.1093/nar/gkz380. 31114893 PMC6602508

[26] Akhter S, Aziz RK, Edwards RA 2012 PhiSpy: a novel algorithm for finding prophages in bacterial genomes that combines similarity- and composition-based strategies. Nucleic Acids Res 40:e126. doi:10.1093/nar/gks406. 22584627 PMC3439882

[27] Bae JS, Da F, Liu R, He L, Lv H, Fisher EL, Rajagopalan G, Li M, Cheung GYC, Otto M 2020 Staphylococcal enterotoxin B contributes to *Staphylococcus aureus* systemic infection. J Infect Dis jiaa584. doi:10.1093/infdis/jiaa584. 32937658 PMC8161638

[28] Gilot P, Lina G, Cochard T, Poutrel B 2002 Analysis of the genetic variability of genes encoding the RNA III-activating components Agr and TRAP in a population of *Staphylococcus aureus* strains isolated from cows with mastitis. J Clin Microbiol 40:4060–4067. doi:10.1128/jcm.40.11.4060-4067.2002. 12409375 PMC139642

[29] Carver T, Thomson N, Bleasby A, Berriman M, Parkhill J 2009 DNAPlotter: circular and linear interactive genome visualization. Bioinformatics 25:119–120. doi:10.1093/bioinformatics/btn578. 18990721 PMC2612626

